# Gastrointestinal Biomarkers and Their Association with Feeding in the First Five Days of Pediatric Critical Illness

**DOI:** 10.1097/MPG.0000000000003950

**Published:** 2023-09-20

**Authors:** Karlien Veldscholte, Jessie M. Hulst, Renate D. Eveleens, Rogier C.J. de Jonge, Barbara A.E. de Koning, Sjoerd A.A. van den Berg, Ronald van der Wal, George J.G. Ruijter, Dimitris Rizopoulos, Ilse Vanhorebeek, Jan Gunst, Michaël Casaer, Greet Van den Berghe, Koen F.M. Joosten, Sascha C.A.T. Verbruggen

**Affiliations:** From the * Department of Neonatal and Pediatric Intensive Care, Division of Pediatric Intensive Care, Erasmus MC Sophia Children’s Hospital, Rotterdam, The Netherlands; the † Department of Pediatrics, University of Toronto, Toronto, Canada; the ‡ Department of Nutritional Sciences, University of Toronto, Toronto, Canada; the § Division of Gastroenterology, Hepatology and Nutrition, The Hospital for Sick Children, Toronto, Canada; the ∥ Department of Anesthesiology, Amsterdam University Medical Centers, AMC, Amsterdam, the Netherlands; ¶ Pediatric Gastroenterology, Erasmus MC Sophia Children’s Hospital, Rotterdam, the Netherlands; the # Department of Clinical Chemistry, Erasmus MC, Rotterdam, The Netherlands; the ** Department of Internal Medicine, Erasmus MC, Rotterdam, The Netherlands; the †† Department of Clinical Genetics, Erasmus MC, Rotterdam, The Netherlands; the ‡‡ Department of Biostatistics, Erasmus MC, Rotterdam, the Netherlands; the §§ Department of Epidemiology, Erasmus MC, Rotterdam, the Netherlands; the ∥∥ Clinical Division and Laboratory of Intensive Care Medicine, Department of Cellular and Molecular Medicine, KU Leuven, Leuven, Belgium.

**Keywords:** enteral nutrition, feeding intolerance, gastrointestinal symptoms, pediatric critical illness

## Abstract

**Objectives:**

Predicting the patients’ tolerance to enteral nutrition (EN) would help clinicians optimize individual nutritional intake. This study investigated the course of several gastrointestinal (GI) biomarkers and their association with EN advancement (ENA) longitudinally during pediatric intensive care unit (PICU) admission.

**Methods:**

This is a secondary analysis of the Early versus Late Parenteral Nutrition in the Pediatric Intensive Care Unit randomized controlled trial. EN was started early and increased gradually. The cholecystokinin (CCK), leptin, glucagon, intestinal fatty acid-binding protein 2 (I-FABP2), and citrulline plasma concentrations were measured upon PICU admission, day 3 and day 5. ENA was defined as kcal EN provided as % of predicted resting energy expenditure. The course of the biomarkers and ENA was examined in patients with samples on all time points using Friedman and Wilcoxon signed-rank tests. The association of ENA with the biomarkers was examined using a 2-part mixed-effects model with data of the complete population, adjusted for possible confounders.

**Results:**

For 172 patients, median age 8.6 years (first quartile; third quartile: 4.2; 13.4), samples were available, of which 55 had samples on all time points. The median ENA was 0 (0; 0) on admission, 14.5 (0.0; 43.8) on day 3, and 28.0 (7.6; 94.8) on day 5. During PICU stay, CCK and I-FABP2 concentrations decreased significantly, whereas glucagon concentrations increased significantly, and leptin and citrulline remained stable. None of the biomarkers was longitudinally associated with ENA.

**Conclusions:**

Based on the current evidence, CCK, leptin, glucagon, I-FABP2, and citrulline appear to have no added value in predicting ENA in the first 5 days of pediatric critical illness.

## Graphical abstract

**Figure:**
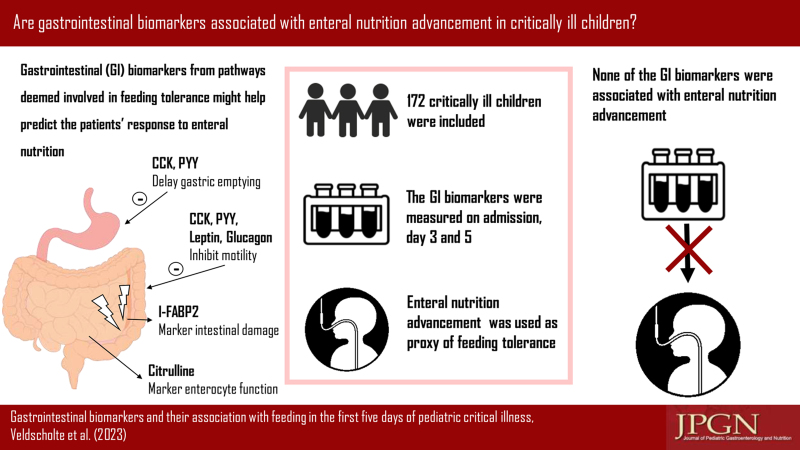

**What Is Known**

- Feeding intolerance (FI) prevents nutritional targets from being reached in critically ill children.
- A laboratory marker for FI is lacking.
- Pathways deemed involved in FI include gastrointestinal dysmotility, damage to enterocytes, and enterocyte function.

**What Is New**

- Cholecystokinin (CCK, dysmotility) and intestinal fatty acid-binding protein 2 (I-FABP2, damage to enterocytes) concentrations decreased during pediatric intensive care unit stay, whereas glucagon (dysmotility) concentrations increased, and leptin (dysmotility) and citrulline (enterocyte function) concentrations remained stable.
- The course of CCK, leptin, glucagon, I-FABP2, and citrulline concentrations were not associated with the enteral nutrition intake as a percentage of predicted resting energy expenditure as a proxy of FI.

Predicting the patients’ tolerance to enteral nutrition (EN) would help clinicians optimize individual nutritional intake and potentially clinical outcomes (1,2). Biomarkers that reflect the pathophysiological pathways of feeding intolerance (FI) might help diagnose FI (2). Pathways hypothesized involved in FI are gastrointestinal (GI) dysmotility, enterocyte damage, and impaired enterocyte function.

So far, results of studies examining the usefulness of biomarkers in the prediction of FI in critically ill patients have been conflicting (2–7). Only 4 studies have been conducted in critically ill children, with relatively small sample sizes (4–7). Moreover, only 1 study (7) (performed in children with congenital heart disease) has investigated the association of biomarkers and FI longitudinally. Examining solely single time points disregards the dynamics of the course of illness and the biomarkers (2).

Cholecystokinin (CCK) and peptide-YY (PYY) are both gut hormones and markers for GI dysmotility, as they both inhibit GI motility and delay gastric emptying (8–11). Leptin and glucagon also inhibit GI motility (12,13). All 4 GI dysmotility markers are expected to be high in FI. Regarding enterocyte damage, intestinal fatty acid-binding protein 2 (I-FABP2) is released when the intestinal mucosa is impaired (14,15) and is therefore expected to be positively associated with FI. Citrulline is a non-essential amino acid synthesized and released by small intestinal enterocytes (16). As low citrulline concentrations indicate poor enterocyte function, the citrulline concentrations are expected to be low in FI.

We hypothesized that CCK, PYY, leptin, glucagon, I-FABP2 and citrulline might be useful in predicting EN advancement (ENA) and FI symptoms (FIS) in critically ill children. This study aimed to investigate the course of these GI biomarkers and their association with ENA longitudinally in the first 5 days of pediatric critical illness.

## METHODS

### Study Design and Participants

This is a secondary analysis of the multicenter Early versus Late Parenteral Nutrition in the Pediatric Intensive Care Unit (PEPaNIC) randomized controlled trial (RCT) (ClinicalTrials.gov: NCT01536275). The study protocol and the trial results have been published previously (17,18). In brief, critically ill children (term neonate to 17 years old) who were expected to stay in the pediatric intensive care unit (PICU) for at least 24 hours and who had a medium-to-high risk of malnutrition assessed via the Screening Tool for Risk on Nutritional Status and Growth score (STRONGkids) (19) were eligible for participation in the PEPaNIC trial. From June 18, 2012, through July 27, 2015, 1440 critically ill children were included and randomized into 2 groups. The control group received supplemental parenteral nutrition (PN) within 24 hours of admission to the PICU (early-PN), whereas the intervention group received supplemental PN only after 1 week of admission if EN was insufficient (late-PN). In both groups, EN was started as soon as possible. EN was provided via tube feeding and increased gradually according to the local protocol at the discretion of the treating physician. The caloric target was based on the body weight and calculated with the Schofield equation (20), aiming for up to 200% of predicted resting energy expenditure (pREE) in neonates and declining to 130% of pREE in adolescents. The protocol allowed half EN on the first day and full EN from the second day onwards. Per protocol, the caloric intake was the same for patients of all diagnoses. Protein and energy-enriched formulas or fortified human milk were provided to patients with fluid restriction or mechanical ventilation. The EN intake was similar for both randomization groups (17). Patients from both randomization groups received intravenous micronutrients (vitamins, electrolytes, and trace elements) from day 2 until EN provided >80% of the caloric target, either via PN or intravenous fluids (21). Blood samples for this secondary analysis were collected only in Rotterdam, in case a sufficient amount of blood could be taken after the primary study samples, according to ethical guidelines.

### Data Collection

For all patients, daily records regarding nutritional intake, laboratory analysis results, GI symptoms, and procedures and treatments were collected during the intervention period. ENA was defined as % caloric needs based on pREE, provided by EN (%EN of pREE). FIS were defined as a large gastric residual volume (≥50% of delivered EN over 24 hours) or vomiting or aspiration and were recorded daily.

### Blood Samples

Blood samples were collected in ethylenediaminetetraacetic acid (EDTA) tubes upon PICU admission (day 0 or 1) and on days 3 and 5 if patients were still participating in the study by that time. The blood samples were processed for plasma collection by centrifugation, after which they were stored at −80°C. Plasma concentrations of CCK, PYY, leptin, glucagon, and I-FABP2 were assessed with enzyme-linked immunosorbent assays (ELISA) according to the manufacturer’s protocol (CCK: Cloud-Cone Corp. CEA802Ca; PYY: Cloud-Cone Corp. CEB067Hu; leptin: Mediagnost ELISA for leptin E07; glucagon: Mercodia 10-1271-01; I-FABP2: BioVendor RD191246200R). Citrulline concentrations were assessed using liquid chromatography-mass spectrometry. The assays were measured in batches sorted by day. For results below the measuring range (limit of quantitation, LOQ), the values for I-FABP2 concentrations were extrapolated using the calibration curve. For the other biomarkers, the values were estimated using LOQ/√2.

### Statistical Analysis

Continuous data were reported as the mean and standard deviation (SD) or as the median and first quartile (Q1); third quartile (Q3) (Q1; Q3), as appropriate. Categorical data were reported as numbers and percentages. Data analyses were performed in R Statistical Software version 4.1.2 (R core team 2021). The complete reference list of the used R packages is shown in Method 1, Supplemental Digital Content 1, http://links.lww.com/MPG/D320.

The primary outcome was the course of the biomarker concentrations during PICU stay. The course was studied only in patients with available biomarker concentrations on all days (admission, day 3, and day 5). The course was displayed in graphs, and the Friedman and (post hoc) Wilcoxon signed-rank tests were used to examine the statistical significance of differences in biomarker concentrations throughout PICU stay.

The secondary outcome was the longitudinal association between the biomarker concentrations and ENA. To investigate this association, a 2-part mixed effects model was used. This model combines a mixed-effects logistic regression for the dichotomous outcome zero or positive EN intake and a linear mixed-effects sub-model for the natural logarithm of the positive EN intake measurements. For both sub-models, the random-effects structure was random intercepts. In the fixed-effects part of the linear mixed model, we included the main effects of the follow-up time variable, diagnostic group, Pediatric Logistic Organ Dysfunction (PELOD) score (22), the use of inotropic agents and post-pyloric feeding (as compared to gastric feeding), and the biomarkers. The covariates were chosen based on a previous secondary analysis of the PEPaNIC RCT investigating the association of patient characteristics and EN intake (23). For the 2-part mixed effects model, the data of the full study population (n = 172) was included, and multiple imputation was used to impute missing covariate information. The variables were transformed if necessary to perform the analysis properly. A more detailed description of the 2-part mixed-effects model is in Method 2, Supplemental Digital Content 2, http://links.lww.com/MPG/D321.

As additional analyses, the differences in biomarker concentrations per day between patients with FIS and without FIS, and between patients in the early-PN and the late-PN group, were investigated using the Mann-Whitney *U* test.

## RESULTS

**FIGURE 1.**
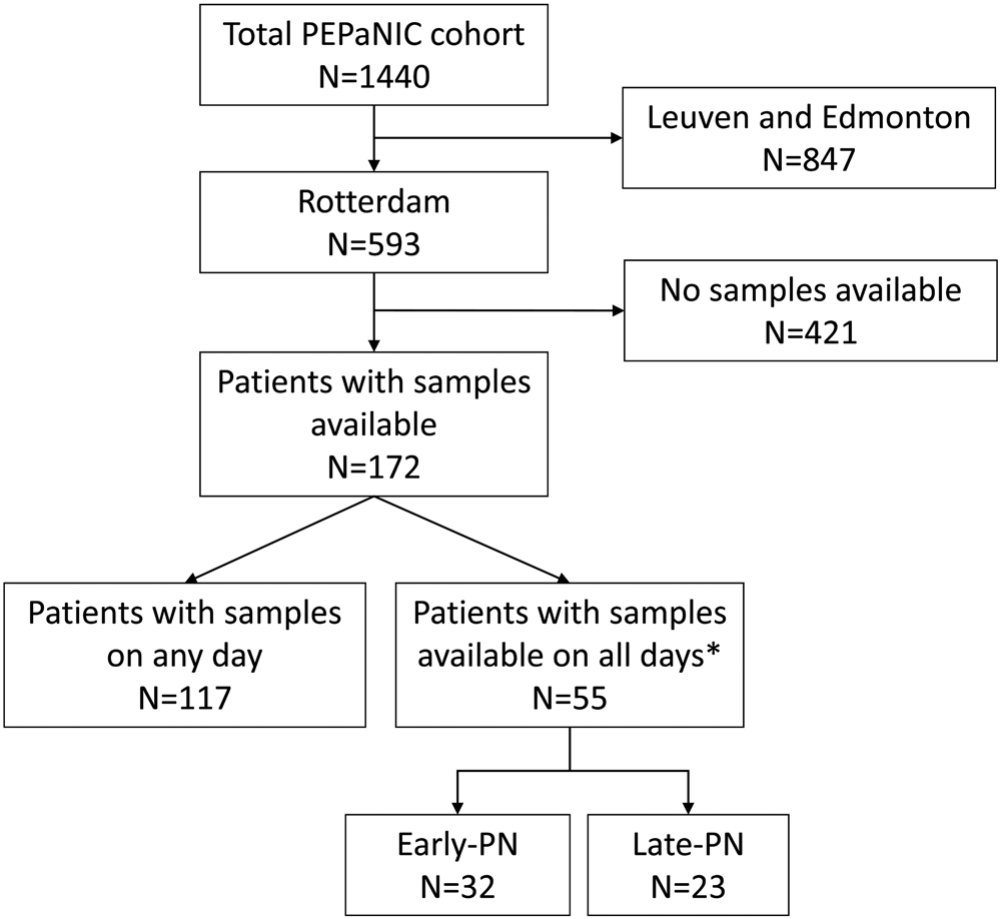
Flowchart for inclusion of patients. *Admission (day 0 or 1), day 3, and day 5. PEPaNIC = Early versus Late Parenteral Nutrition in the Pediatric Intensive Care Unit; PN = parenteral nutrition.

A total of 172 patients (85 early-PN and 87 late-PN) were included in this secondary analysis, of which 94 were still participating in the study on day 3 and 66 on day 5. For 55 patients (32 early-PN and 23 late-PN), samples were available on all days (admission, day three and five) (Fig. 1). Baseline characteristics of the complete study population of this secondary analysis and of the subgroup with samples on all studied days are shown in Table 1.

**TABLE 1.** Baseline characteristics

Characteristic	PEPaNIC population (N = 1440)	Population of this secondary analysis (N = 172)	Patients with samples on all days (N = 55)
Randomization group: Early-PN	723 (50%)	85 (49%)	32 (58%)
Age, y	1.5 (0.3; 6.3)	8.6 (4.2; 13.3)	11.2 (6.3; 14.4)
Sex: male	830 (58%)	103 (60%)	29 (53%)
STRONGkids category: high risk	152 (11%)	17 (10%)	5 (9%)
Diagnostic group			
Surgical			
Cardiac	547 (38%)	18 (10%)	4 (7%)
Other	426 (30%)	70 (41%)	27 (49%)
Medical			
Neurologic	103 (7%)	16 (9%)	6 (11%)
Other	364 (25%)	68 (40%)	18 (33%)
PIM3 score	−3.5 (−4.4; −2.4)	−2.6 (−3.6; −1.2)	−2.3 (−3.0; −0.7)

Data are no. (%) or median (Q1; Q3). PEPaNIC = Early versus Late Parenteral Nutrition in the Pediatric Intensive Care Unit; PIM3 = Pediatric Index of Mortality; PN = parenteral nutrition; STRONGkids = Screening Tool for Risk on Nutritional Status and Growth score.

**FIGURE 2.**
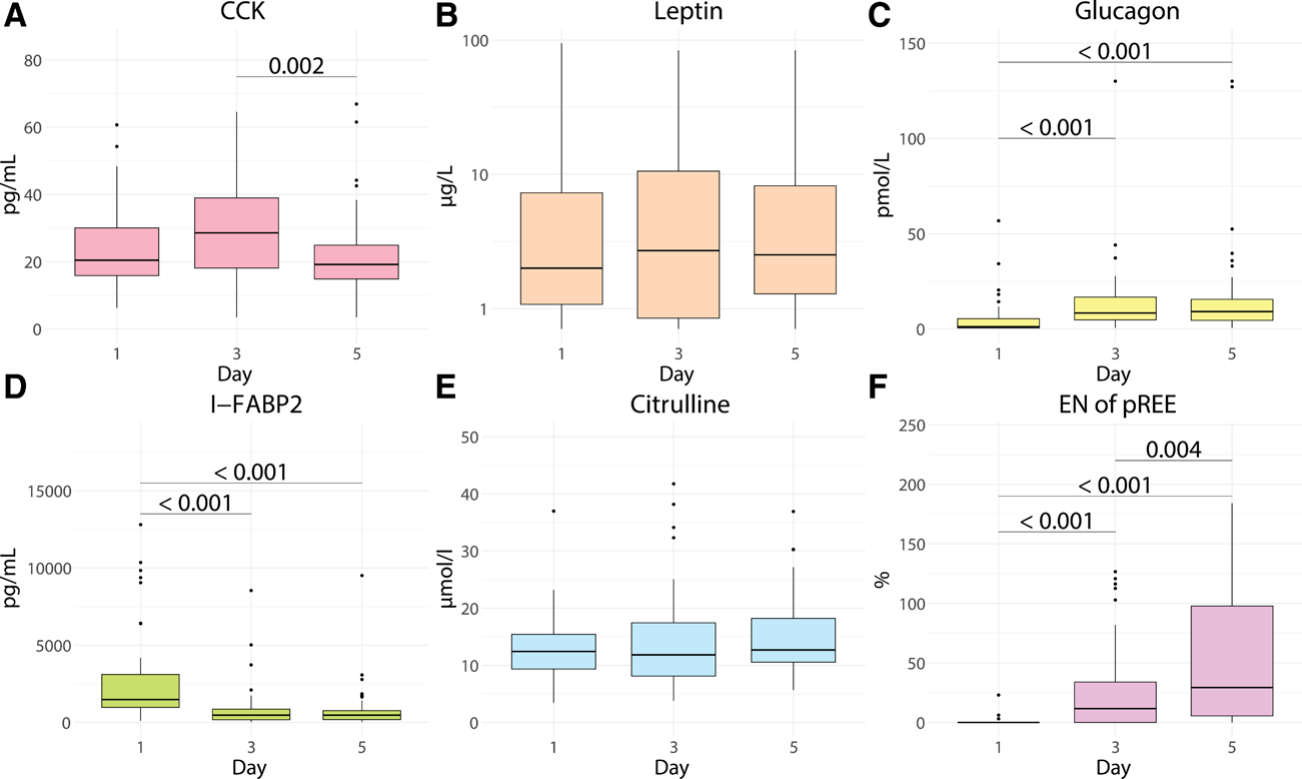
Boxplots for the course of (A) CCK, (B) leptin, (C) glucagon, (D) I-FABP2, (E) citrulline, and (F) enteral intake for patients with samples on all days (n = 55). CCK = cholecystokinin; EN = enteral nutrition; I-FABP2 = intestinal fatty-acid binding protein 2; pREE = predicted resting energy expenditure.

The course of the biomarkers and ENA for the 55 patients with samples available upon admission, day 3, and 5 are shown in Figure 2. The enteral intake increased significantly during the first 5 days (Fig. 2F), as in line with the feeding protocol. The concentrations of CCK, glucagon, and I-FABP2 changed significantly during PICU stay (Fig. 2); CCK concentrations decreased on day 5 in comparison to day 3 (Fig. 2A), glucagon concentrations increased on day 3 and day 5 in comparison to admission (Fig. 2C), and I-FABP2 concentrations decreased on day 3 and day 5 in comparison to admission (Fig. 2D). No statistically significant differences were apparent for leptin and citrulline concentrations (Fig. 2B and E). The results for PYY are shown in the supplemental files because of uncertainty regarding the representativeness of the measured concentrations (Figure 1A, Supplemental Digital Content 3, http://links.lww.com/MPG/D322). The biomarker concentrations and %EN of pREE per day for all 172 patients, including patients with samples on only 1 or 2 days, are shown in Table 1, Supplemental Digital Content 4, http://links.lww.com/MPG/D323.

The results of the longitudinal analysis regarding the association of biomarkers with ENA in the complete study population (n = 172) are shown in Table 2. None of the biomarkers was significantly associated with ENA when adjusted for possible confounders (diagnosis, daily PELOD score, the use of inotropic agents, and post-pyloric feeding vs nasogastric feeding). Method 2, Supplemental Digital Content 2, http://links.lww.com/MPG/D321 provides a more detailed explanation of the interpretation of the results.

**TABLE 2.** The longitudinal associations of %EN of pREE and the biomarkers, corrected for possible confounders, tested with 2-part mixed effects models.

	CCK			Leptin			Glucagon		
	Exp(coef)	95% CI	*P* value	Exp(coef)	95% CI	*P* value	Exp(coef)	95% CI	*P* value
Intercept	0.15	0.07–0.32		0.14	0.07–0.25		0.15	0.08–0.27	
Log2(biomarker)	1.00	0.86–1.17	0.96	1.09	0.98–1.20	0.12	1.01	0.89–1.14	0.88
Time, d	1.29	1.09–1.52	0.003	1.28	1.09-1.50	0.003	1.28	1.09–1.51	0.003
Diagnosis—Surgery	1.11	0.29–4.31	0.88	1.09	0.27–4.36	0.90	1.12	0.29-4.28	0.86
Diagnosis—Neurological	1.31	0.65–2.63	0.45	1.24	0.64–2.43	0.52	1.31	0.65–2.63	0.45
Diagnosis—Respiratory	1.33	0.82–2.16	0.25	1.24	0.76–2.01	0.39	1.33	0.82–2.15	0.24
PELOD score	0.98	0.97–1.00	0.04	0.98	0.97–1.00	0.026	0.98	0.97–1.00	0.031
Inotropic agents	0.67	0.46–0.99	0.05	0.66	0.45–0.96	0.032	0.67	0.45–1.00	0.048
Post-pyloric feeding	1.36	0.92–1.17	0.12	1.37	0.98–1.20	0.12	1.38	0.93–2.04	0.11
	I-FABP2			Citrullin			PYY		
	Exp(coef)	95% CI	*P* value	Exp(coef)	95% CI	*P* value	Exp(coef)	95% CI	*P* value
Intercept	0.14	0.07–0.28		0.11	0.05–0.26		0.13	0.07–0.26	
Log2(biomarker)	1.02	0.94–1.10	0.70	1.10	0.90–1.33	0.36	1.07	0.87–1.30	0.53
Time, d	1.27	1.06–1.53	0.01	1.25	1.04–1.50	0.017	1.26	1.05–1.52	0.015
Diagnosis–Surgery	1.10	0.28–4.27	0.89	0.13	0.29–4.37	0.86	1.14	0.29–4.41	0.85
Diagnosis—Neurological	1.30	0.65–2.62	0.46	0.29	0.64–2.57	0.48	1.31	0.65–2.63	0.44
Diagnosis—Respiratory	1.33	0.83–2.16	0.24	1.35	0.84–2.19	0.22	1.30	0.80–2.14	0.29
PELOD score	0.98	0.97–1.00	0.027	0.98	0.97–1.00	0.038	0.98	0.97–1.00	0.027
Inotropic agents	0.67	0.46–0.98	0.040	0.68	0.46–1.01	0.05	0.67	0.45–0.99	0.044
Post-pyloric feeding	1.35	0.92–1.99	0.13	1.34	0.92–1.96	0.13	1.36	0.92–1.99	0.12

The reported coefficients, and the corresponding 95% confidence intervals and *P* values are based on the marginal mean. The exponents of the coefficients are in the original scale of the main outcome. Hence, the exponent of the coefficients quantifies the multiplicative increase in the average of the main outcome. A unit increase for the log2(biomarker) corresponds to a doubling of the biomarker levels on the original scale. 95% CI = 95% confidence interval; CCK = cholecystokinin; d = day; I-FABP2 = intestinal fatty-acid binding protein; PELOD = Pediatric Logistic Organ Dysfunction; PYY = peptide-YY.

FIS occurred in 19 of the 94 patients (20%) on day 3, and in 14 of the 66 patients (21%) on day 5. Patients with FIS had significantly lower CCK concentrations on day 3 than patients without FIS [19.9 pg/mL (14.0; 28.2) vs 30.2 pg/mL (22.2; 40.0), *P* = 0.012, Table 2, Supplemental Digital Content 5, http://links.lww.com/MPG/D324]. The concentrations of other biomarkers on day 3 and of all biomarkers on day 5 (including CCK) were not significantly different between patients with or without FIS (Table 2, Supplemental Digital Content 5, http://links.lww.com/MPG/D324). The enteral intake did not differ between patients with or without FIS (Table 2, Supplemental Digital Content 5, http://links.lww.com/MPG/D324). Baseline characteristics of patients who suffered from FIS on day 3 or day 5 and patients who never had FIS are shown in Table 3, Supplemental Digital Content 6, http://links.lww.com/MPG/D325. None of the patients with abdominal surgery had FIS, and 39% of the patients with FIS were neurosurgery patients (Table 3, Supplemental Digital Content 6, http://links.lww.com/MPG/D325). Although the intake did not differ between patients with or without FIS, on day 3 none of the abdominal surgery patients had any intake, and on day 5 only one of the abdominal surgery patients had nutritional intake (20% of pREE). GI biomarker concentrations did not differ between patients in the early-PN and late-PN group (Figure 2, Supplemental Digital Content 7, http://links.lww.com/MPG/D326 and Figure 1B, Supplemental Digital Content 3, http://links.lww.com/MPG/D322).

## DISCUSSION

This study investigated the course of several GI biomarkers during the first 5 days of pediatric critical illness and its association with ENA. Whereas leptin and citrulline did not change over time, we found that plasma CCK and I-FABP2 concentrations decreased significantly, and glucagon concentrations increased significantly during the first 5 days of PICU stay. None of the GI biomarkers were longitudinally associated with the amount of EN provided. Moreover, except for lower CCK levels in patients with FIS on day 3, GI biomarker concentrations did not differ between patients with or without FIS. For now, we thus conclude that these biomarkers could not be used as a predictor for ENA during the first 5 days of their PICU stay.

We have studied the course of biomarkers of 3 pathophysiological pathways deemed involved in FI, that is, GI motility, enterocyte damage, and enterocyte function.

In our study, CCK concentrations were lower on day 5 compared to day 3 (Fig. 2A), contrary to a study in critically ill adults that had found an increase during ICU stay (24). Especially because the EN intake was higher on day 5 and CCK is released in response to feeding, we would have expected higher CCK concentrations (10). Nevertheless, as a previous study had shown an exaggerated response to feeding in intolerant patients, the lower CCK concentrations might also reflect a better tolerance (25). CCK concentrations were lower in patients with FIS than those without FIS on day 3 (Table 2, Supplemental Digital Content 5, http://links.lww.com/MPG/D324), which was also the opposite of what we had expected. Nevertheless, the concentrations in our study were within the limits of reference values in healthy infants on all days and for both patients with and without FIS (26,27).

Our results regarding glucagon correspond to a study in critically ill adults that had also found normal glucagon concentrations upon admission, followed by an increase during ICU stay (Fig. 2C) (28). They found that plasma glucagon concentrations were not affected by glucose and insulin infusions, but that they further increased with the administration of PN containing amino acids (28). However, we did not find an association with enteral nutritional intake (Table 2). The overall range of glucagon concentrations was in line with other studies in critically ill children (4) and adults (28,29).

The I-FABP2 concentrations on admission in our study are higher than reference concentrations in healthy children (7). However, Derikx et al (30) also detected high concentrations of I-FABP2 in children with meningococcal sepsis on admission (median 298 pg/mL, range 25–4351 pg/mL), which rapidly declined in survivors during PICU stay, similar to the decrease on day 3 and 5 that we found (Fig. 2D). We hypothesize that the normalization of I-FABP2 concentrations during PICU stay could at least partially be explained by recovery of the intestinal mucosa after the initial “hit” of critical illness.

The range of leptin concentrations was in line with ranges found in critically ill adults (31,32) and healthy children (33). One of the studies in critically ill adults found increasing leptin concentrations during ICU stay (32), whereas we (Fig. 2B) and the other study in critically ill adults found stable concentrations. Nevertheless, the study included only patients with respiratory diagnoses, and the increase was only found in non-septic patients. We have not investigated the course in septic and non-septic patients separately.

Regarding citrulline, a study in children undergoing heart surgery found a decrease in citrulline concentrations post-surgery (7). We found that citrulline concentrations did not change over time (Fig. 2E), with values comparable to the other study’s pre-surgery and post-surgery concentrations (7) and reference ranges in healthy children (34). Although the median leptin and citrulline concentrations did not change over time, a dispersion of measured concentrations could be noted, indicating inter-individual differences.

PYY concentrations increased significantly during the first 5 days of PICU stay (Figure 1, Supplemental Digital Content 3, http://links.lww.com/MPG/D322). However, the measured concentrations of PYY were much lower than concentrations in other studies (4,6,35–37). The differences in analysis techniques do not suffice to explain a discrepancy of this extent. The manufacturer’s protocol was always adhered to, and no technical problems occurred. Although the manufacturer’s protocol of the used assay does not describe the necessity of adding a protease inhibitor, the protocols of other PYY assays do advise this. It remains uncertain if the low concentrations found in our study are a realistic reflection of our patient population, or due to a measurement error, for example, because of degradation.

Despite the variation in the biomarker concentrations, both intra-individually and inter-individually, none of the biomarkers was associated with ENA as a proxy of FI (Table 2). Furthermore, except for lower CCK concentrations in patients with FIS on day 3, GI biomarkers concentrations were not different in patients with or without FIS (Table 2, Supplemental Digital Content 5, http://links.lww.com/MPG/D324). Remarkably, none of the abdominal surgery patients had FIS on day 3 or 5, which might be at least partly related to their very limited intake. Several differences between previous studies (4,6,7) and the current study complicate direct comparison, especially the variation in the outcome variable, that is, the definition of FI or FIS. It is doubtful whether the various clinical parameters used to describe FI all measure the same entity. The debate regarding the correct definition of FI is ongoing, especially since the reliability of the widely used criterion of a large gastric residual volume (GRV) is currently challenged (38). Differences in the measuring methods of biomarkers, patient populations, and the amount of EN provided hamper the direct comparison between studies as well. As we found in our study, also the timing of the measurements in previous studies might have affected the interpretation of the results.

The studied biomarkers are all part of complex pathways and are affected by multiple physiological, pathophysiological, and iatrogenic processes. For example, a study in critically ill adults found a correlation between glucagon and severity of illness (28). Likewise, the biomarker concentrations might rather reflect, for example, the severity of illness. Furthermore, FI is a manifestation of a very complex interplay of clinical features as well as patient management (39,40). It may be too simplistic to aim for parameters such as the studied biomarkers alone to predict FI and guide feeding advancement (2).

Our study has several strengths, of which the longitudinal aspect is unique in this patient population. Secondly, we have investigated multiple GI biomarkers involved in different pathways in an attempt to capture the various aspects of FI. However, our study also has some limitations that need to be addressed. Most importantly, several details regarding GI symptoms and reasons for withholding or not providing EN according to the protocol (eg, fluid restrictions and fasting for procedures) were lacking. Hence, we could not use the proposed definition of FI of Eveleens et al (39), which was developed after this RCT was finished, and combines the inability to reach nutritional targets and specific GI symptoms. We therefore used the amount of EN provided as a proxy of FI, which might be less appropriate. Second, because the available literature lacked a sufficient basis for the expected biomarker concentrations, some concentrations fell outside the detection limits of the chosen assays. Third, the biomarkers were measured in batches sorted per day to make comparisons between different patients on the same day possible. However, this batch analysis could be a confounder in comparing concentrations within a patient over several days, such as in the longitudinal analysis. Lastly, patients in our study were relatively old (median 9 years) due to restrictions regarding the amount of blood that could be taken. It is unclear whether age affects the relation of the biomarkers with FI.

## CONCLUSIONS

Our study showed plasma CCK and I-FABP2 concentrations decrease, whereas glucagon concentrations increase significantly during the first 5 days of pediatric critical illness, emphasizing the dynamics of these biomarkers. We did not find a significant association between the studied GI biomarkers and the amount of EN provided as a proxy of FI in the first 5 days of PICU stay. Based on the current evidence, these biomarkers appear to have no added value in predicting ENA in the first 5 days of pediatric critical illness. Studies using other approaches in combination with a clear definition of FI might be warranted to develop a diagnostic tool for feeding tolerance in critically ill children.

## Acknowledgments

The authors thank the children and their parents or guardians for their willingness to participate in the PEPaNIC study. They also acknowledge the research team members that were involved in the execution of the PEPaNIC study. Moreover, they thank Sharmila Panchoe – Ramcharan and Brigitta van de Lang – Born for performing the CCK, glucagon, leptin, I-FABP2, and PYY measurements, and Niels Willemse for performing the measurements of citrulline. Lastly, they thank Milot van Rijt and Chiara Altruda for their input regarding the initial analyses and manuscript drafting during their research internship.

## Supplementary Material


