# Supplementary material for: Gastrointestinal Biomarkers and Their Association with Feeding in the First Five Days of Pediatric Critical Illness
Source: J Pediatr Gastroenterol Nutr. 2023 Sep 20;77(6):811–8. doi: 10.1097/MPG.0000000000003950 (PMC10642702; doi:10.1097/MPG.0000000000003950)
Supplement: Supplementary file 1 [file mpg-77-0811-s001.pdf]

Methods, Supplemental Digital Content 1. R Packages used

foreign: R Core Team (2020). foreign: Read Data Stored by 'Minitab', 'S', 'SAS', 'SPSS', 'Stata', 'Systat', 'Weka', 'dBase', ... R package version 0.8-81. https://CRAN.R-project.org/package=foreign

ggpubr: Alboukadel Kassambara (2020). ggpubr: 'ggplot2' Based Publication Ready Plots. R package version 0.4.0. https://CRAN.R-project.org/package=ggpubr

GLMMadaptive: Dimitris Rizopoulos (2022). GLMMadaptive: Generalized Linear Mixed Models using Adaptive Gaussian Quadrature. R package version 0.8-5. https://CRAN.R-project.org/package=GLMMadaptive

haven: Hadley Wickham and Evan Miller (2021). haven: Import and Export 'SPSS', 'Stata' and 'SAS' Files. R package version 2.4.3. https://CRAN.R-project.org/package=haven

lme4: Douglas Bates, Martin Maechler, Ben Bolker, Steve Walker (2015). Fitting Linear Mixed-Effects Models Using lme4. Journal of Statistical Software, 67(1), 1-48. https://doi:10.18637/jss.v067.i01.

mice: van Buuren S, Groothuis-Oudshoorn K (2011). “mice: Multivariate Imputation by Chained Equations in R.” *Journal of Statistical Software*, 45(3), 1-67. https://doi:10.18637/jss.v045.i03.

nlme: Pinheiro J, Bates D, DebRoy S, Sarkar D, R Core Team (2021). _nlme: Linear and Nonlinear Mixed Effects Models_. R package version 3.1-153, <URL: https://CRAN.R-project.org/package=nlme>.

openxlsx: Philipp Schauberger and Alexander Walker (2022). openxlsx: Read, Write and Edit xlsx Files. R package version 4.2.5.1. https://CRAN.R-project.org/package=openxlsx

readxl: Hadley Wickham and Jennifer Bryan (2019). readxl: Read Excel Files. R package version 1.3.1. https://CRAN.R-project.org/package=readxl

rstatix: Alboukadel Kassambara (2021). rstatix: Pipe-Friendly Framework for Basic Statistical Tests. R package version 0.7.0. https://CRAN.R-project.org/package=rstatix

tidyverse: Wickham et al., (2019). Welcome to the tidyverse. Journal of Open Source Software, 4(43), 1686, https://doi.org/10.21105/joss.01686
