# Supplementary material for: Gastrointestinal Biomarkers and Their Association with Feeding in the First Five Days of Pediatric Critical Illness
Source: J Pediatr Gastroenterol Nutr. 2023 Sep 20;77(6):811–8. doi: 10.1097/MPG.0000000000003950 (PMC10642702; doi:10.1097/MPG.0000000000003950)
Supplement: Supplementary file 2 [file mpg-77-0811-s002.pdf]

Methods, Supplemental Digital Content 2. Additional information regarding the methods and interpretation of the two-part mixed-effects model.

In the posited models, it was specified that the biomarkers have a zero association with enteral nutrition intake at baseline (i.e., follow-up time zero). A separate model was fitted per biomarker. Multiple imputation by chained equations (predictive mean matching method, package “mice” in R), was used to impute missing covariate information, using 30 imputed datasets. Each imputed dataset has been separately analyzed using the two-part mixed-effects model, and the results were pooled using the multiple imputation formulas. The fit of the model was assessed using scaled simulated residuals. No correction for multiple testing has been performed. For interpretation, the results were presented for the marginalized mean, thus based on the average mixture response of the logistic mixed-effects and linear mixed-effects model.

The biomarker values have been log2-transformed because they exhibited a skewed distribution. The reported coefficients, the corresponding 95% confidence intervals (95%-CI), and p-values are for the marginal mean of enteral nutrition intake, so for the average mixture of the logistic mixed-effects and linear mixed-effects model. The exponents of the coefficients are in the original scale of the main outcome. Hence, the exponent of the coefficients quantifies the multiplicative increase in the average of the main outcome (%EN of pREE). For example, if the exponent of the coefficient for time (in days) is 1.29, it means that the average main outcome (%EN of pREE) is increased by 29% for every unit (day) increase of time. A unit increase for the log2(biomarker) corresponds to a doubling of the biomarker levels on the original scale. Thus, if the exponent of the coefficient for the log2(biomarker) is 1.09, it means that if the biomarker doubles in value on a particular day, the average main outcome on that same day increases by 9%.
