# Supplementary figures and images for: Gastrointestinal Biomarkers and Their Association with Feeding in the First Five Days of Pediatric Critical Illness
Source: J Pediatr Gastroenterol Nutr. 2023 Sep 20;77(6):811–8. doi: 10.1097/MPG.0000000000003950 (PMC10642702; doi:10.1097/MPG.0000000000003950)

A: course of PYY

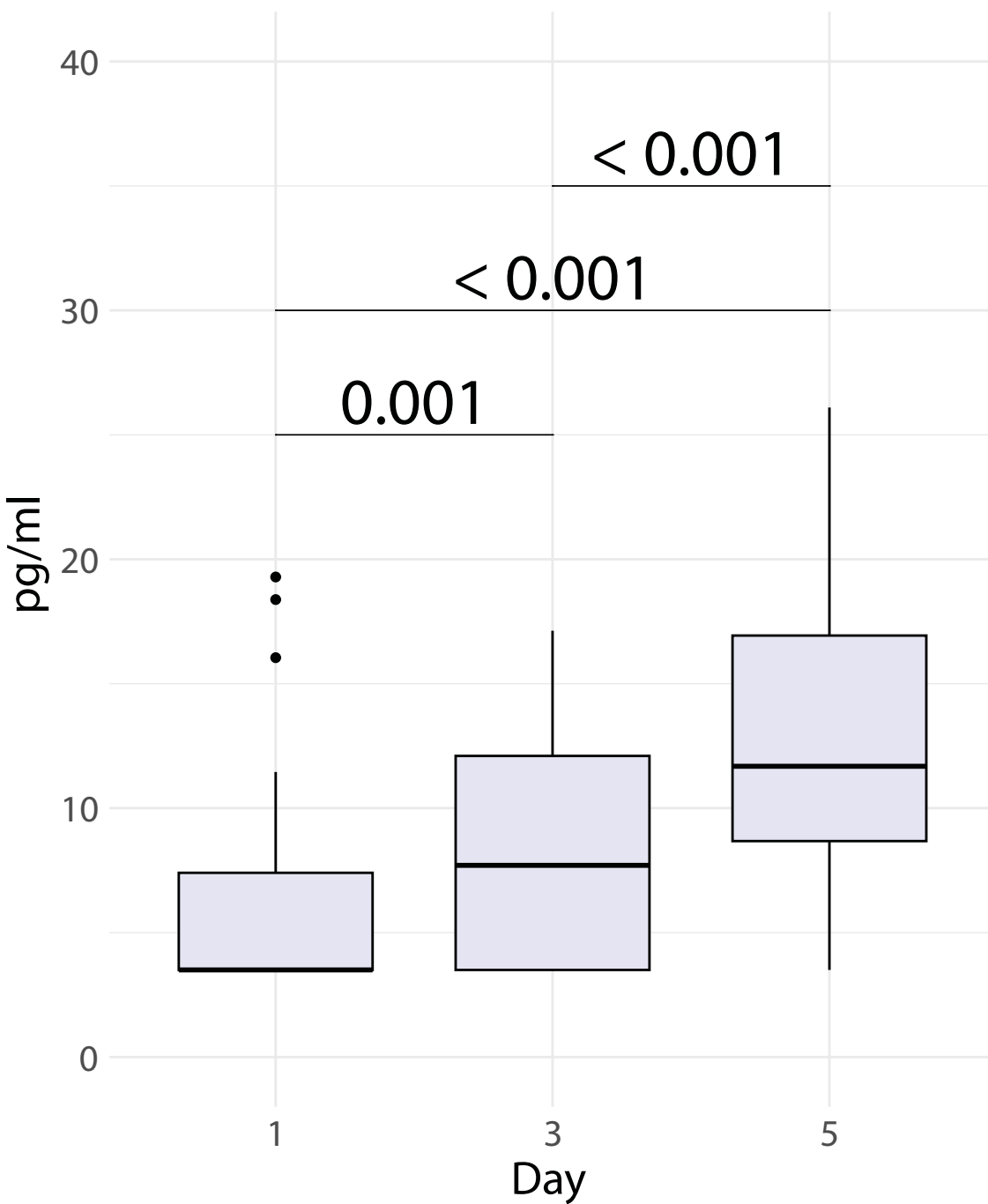

B: PYY early-PN vs. late-PN

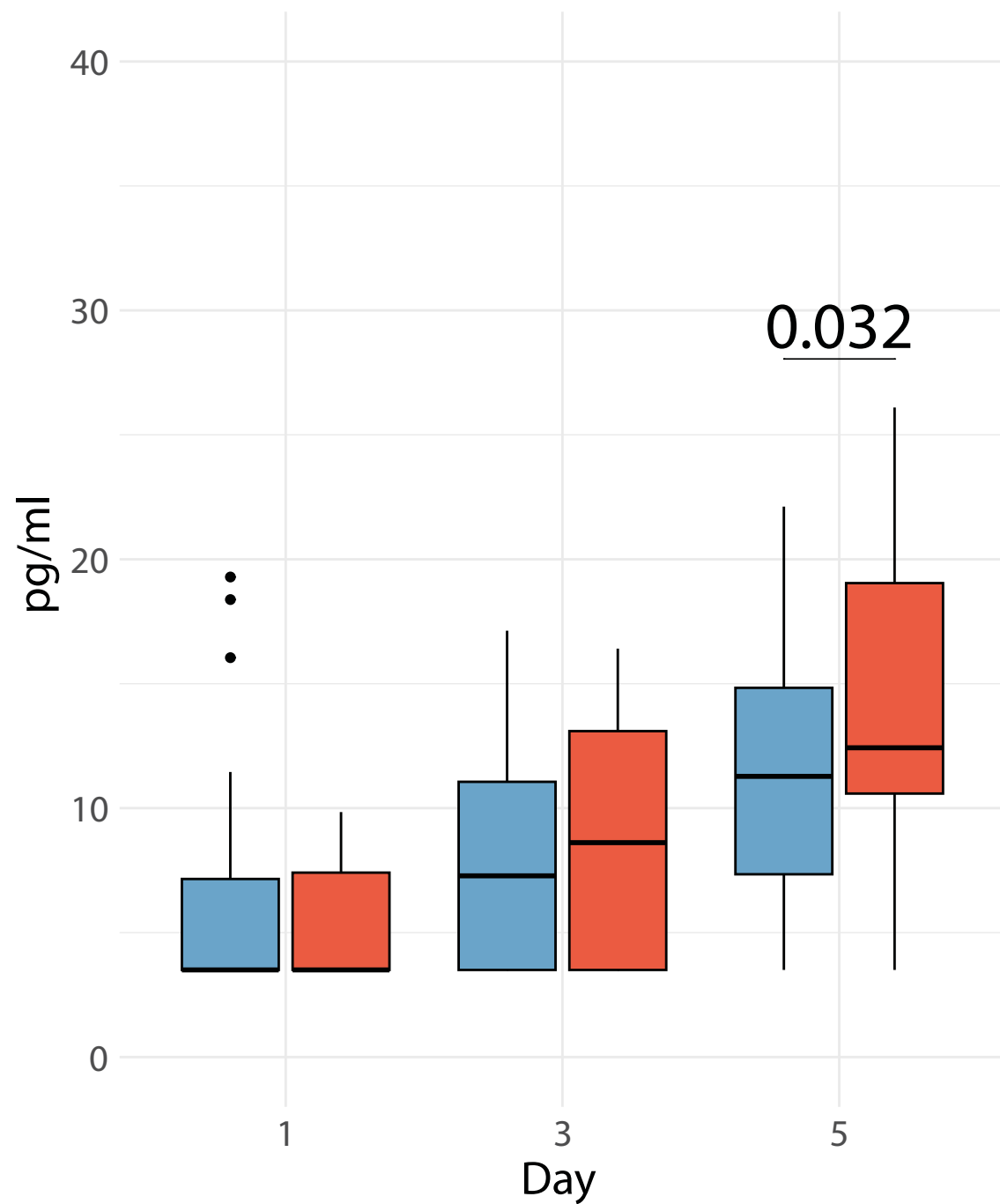

Randomisation    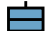 Early-PN    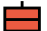 Late-PN

Supplement: Supplementary file 3 [file mpg-77-0811-s003.pdf]

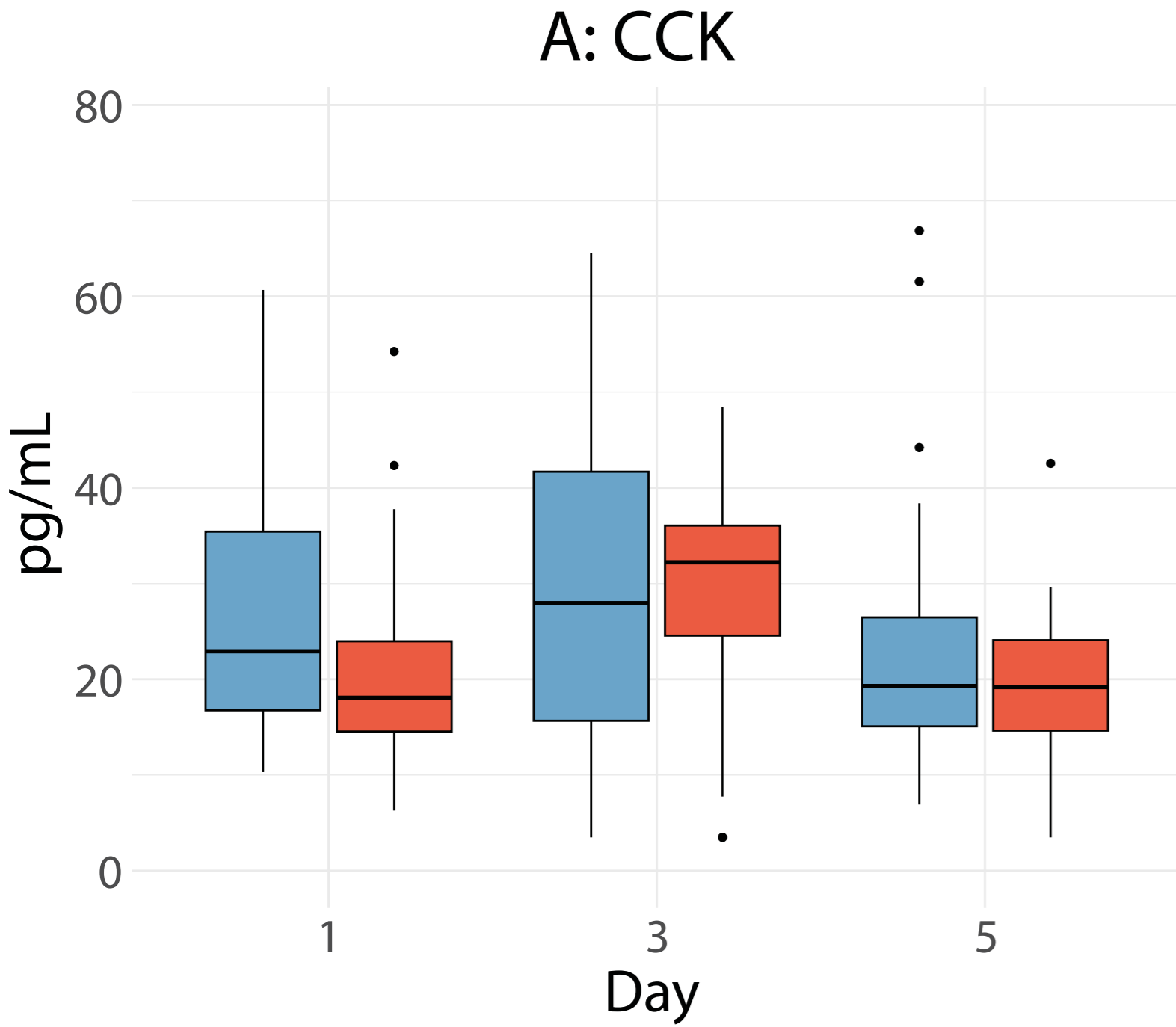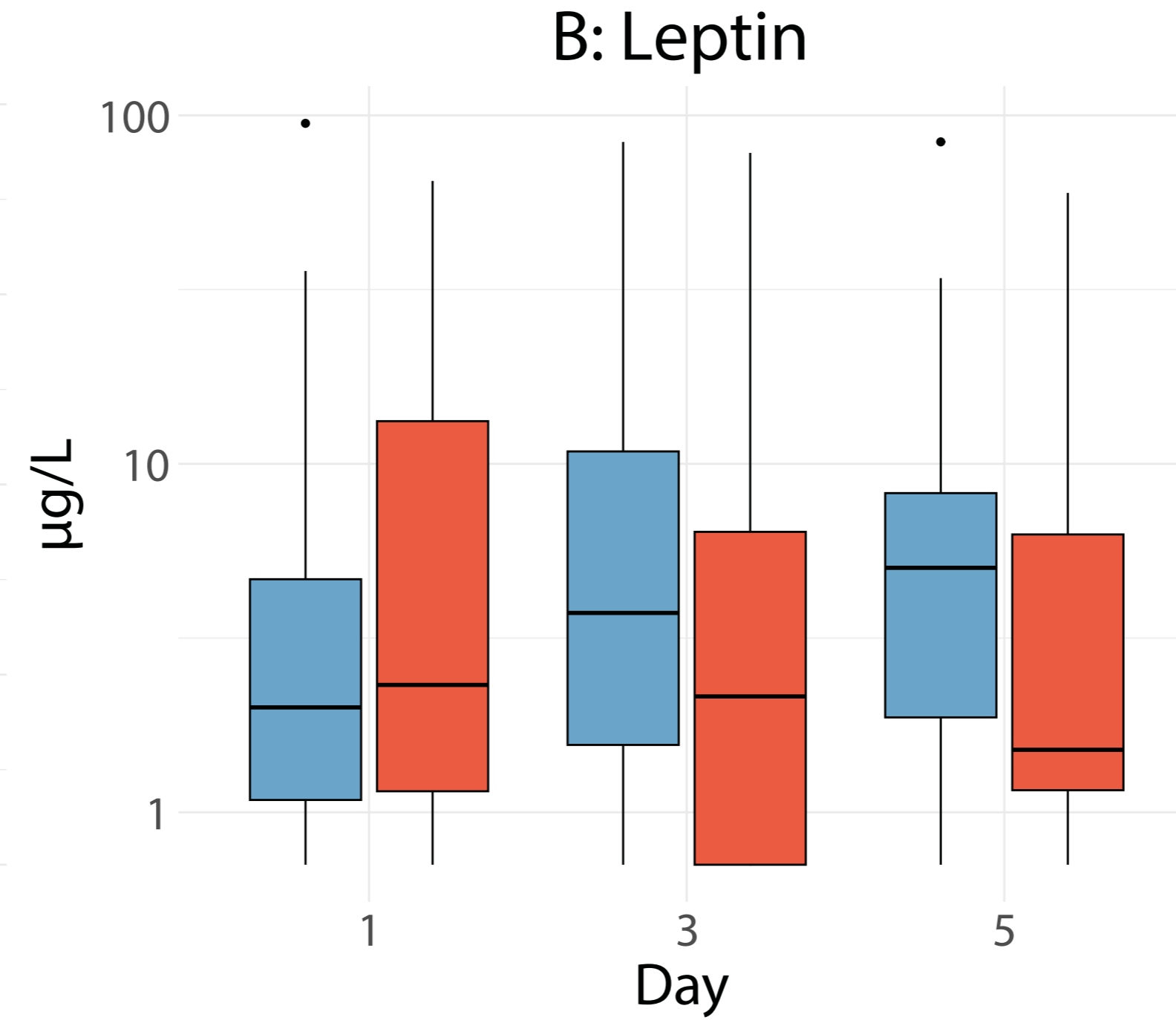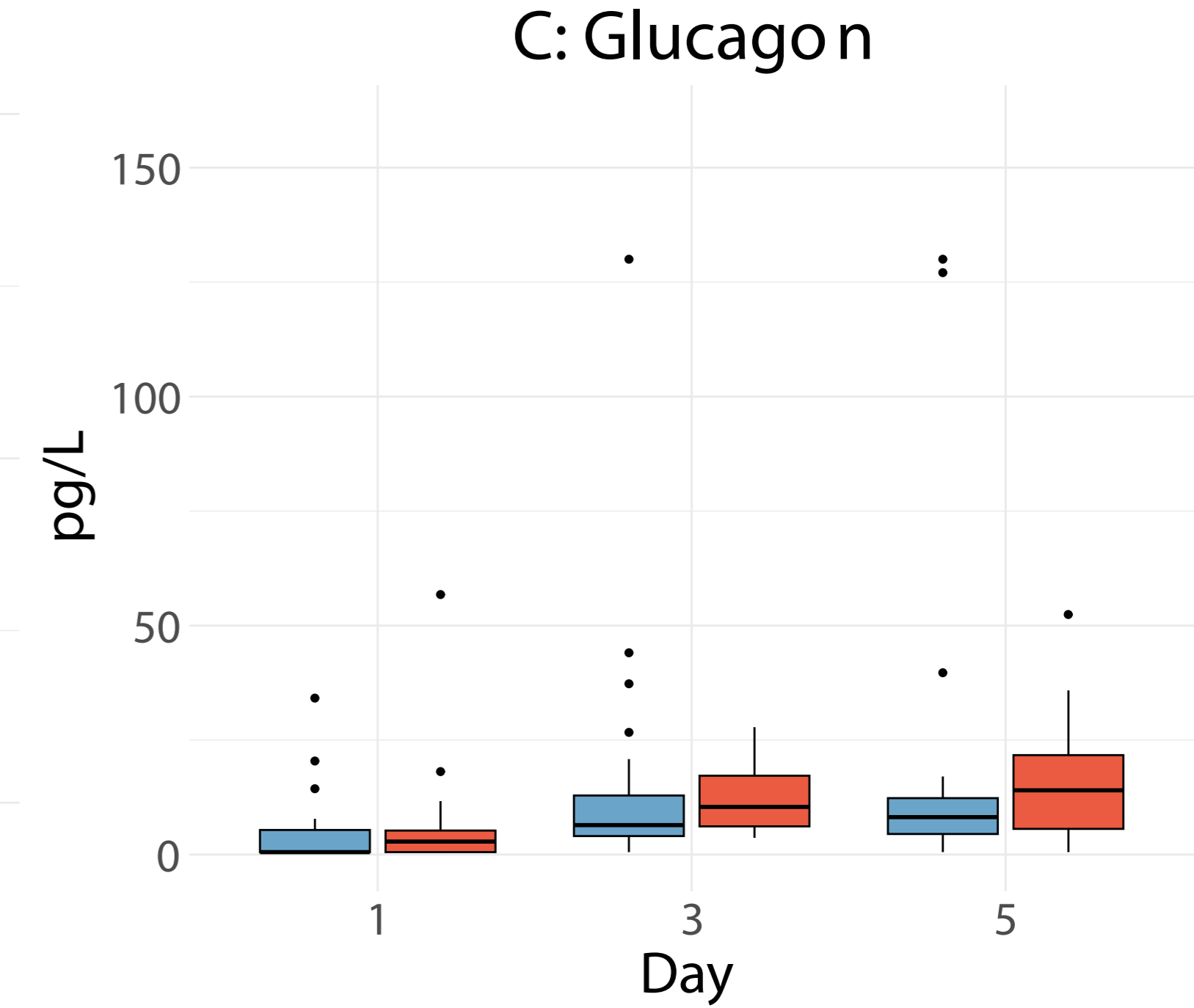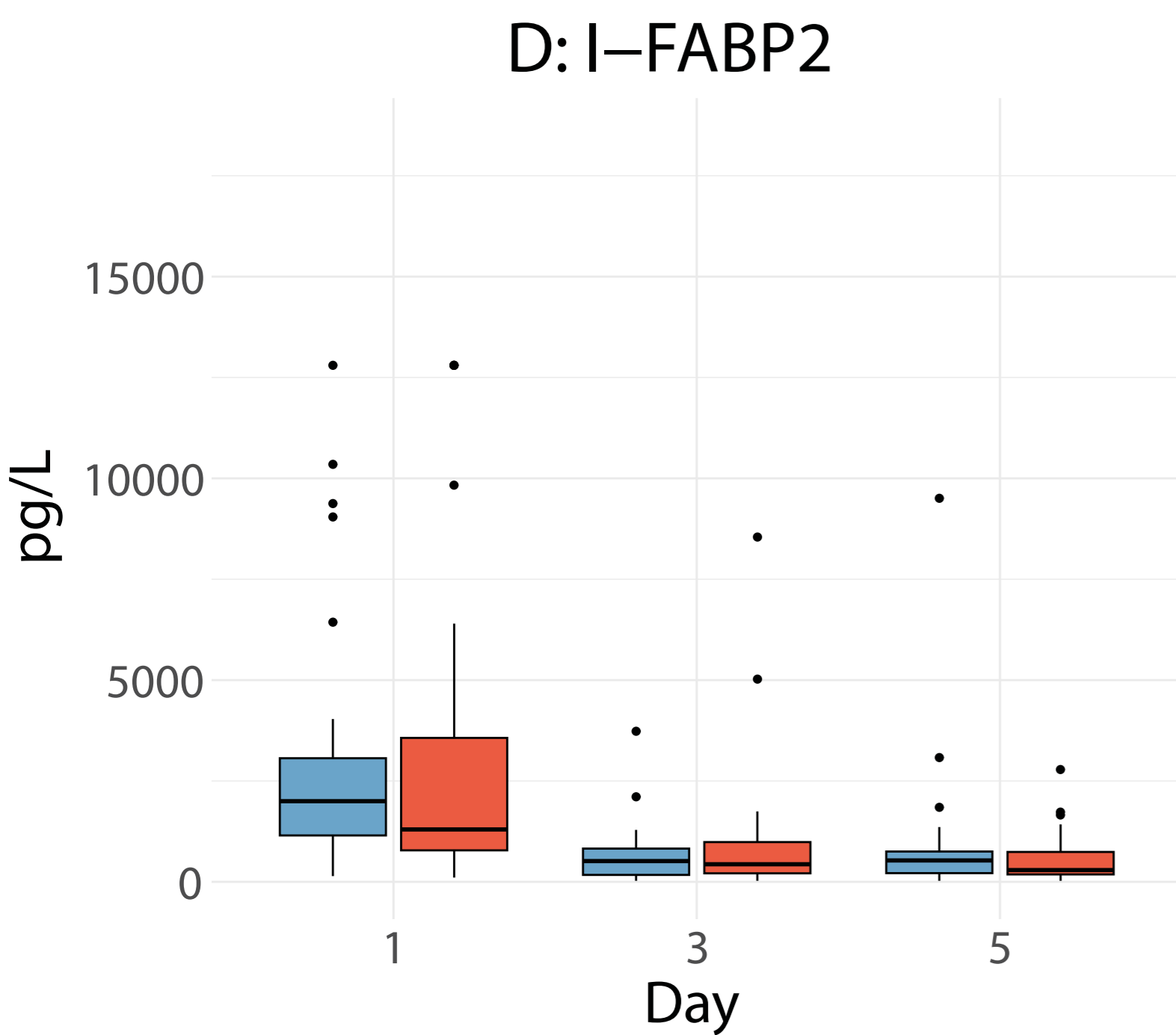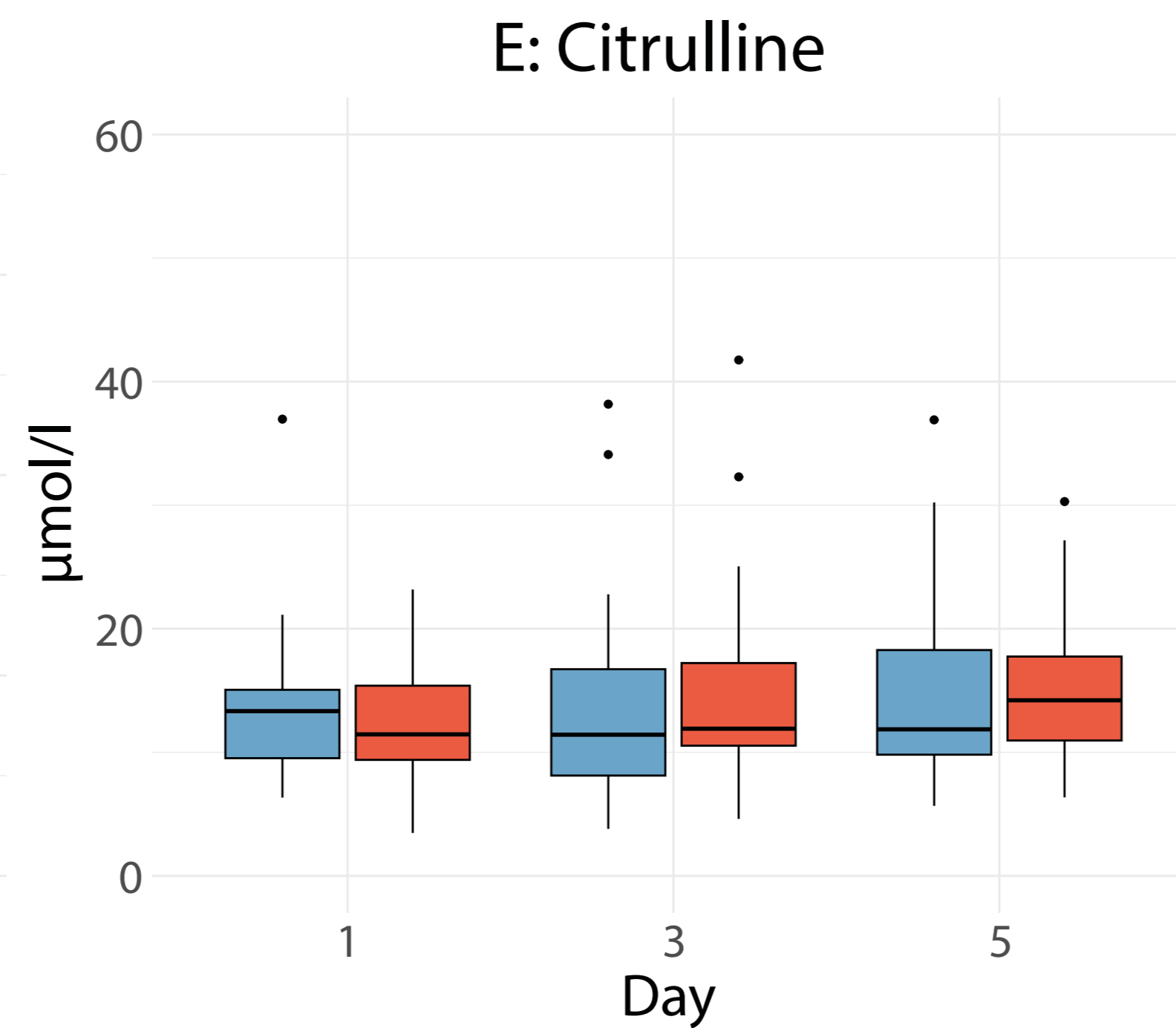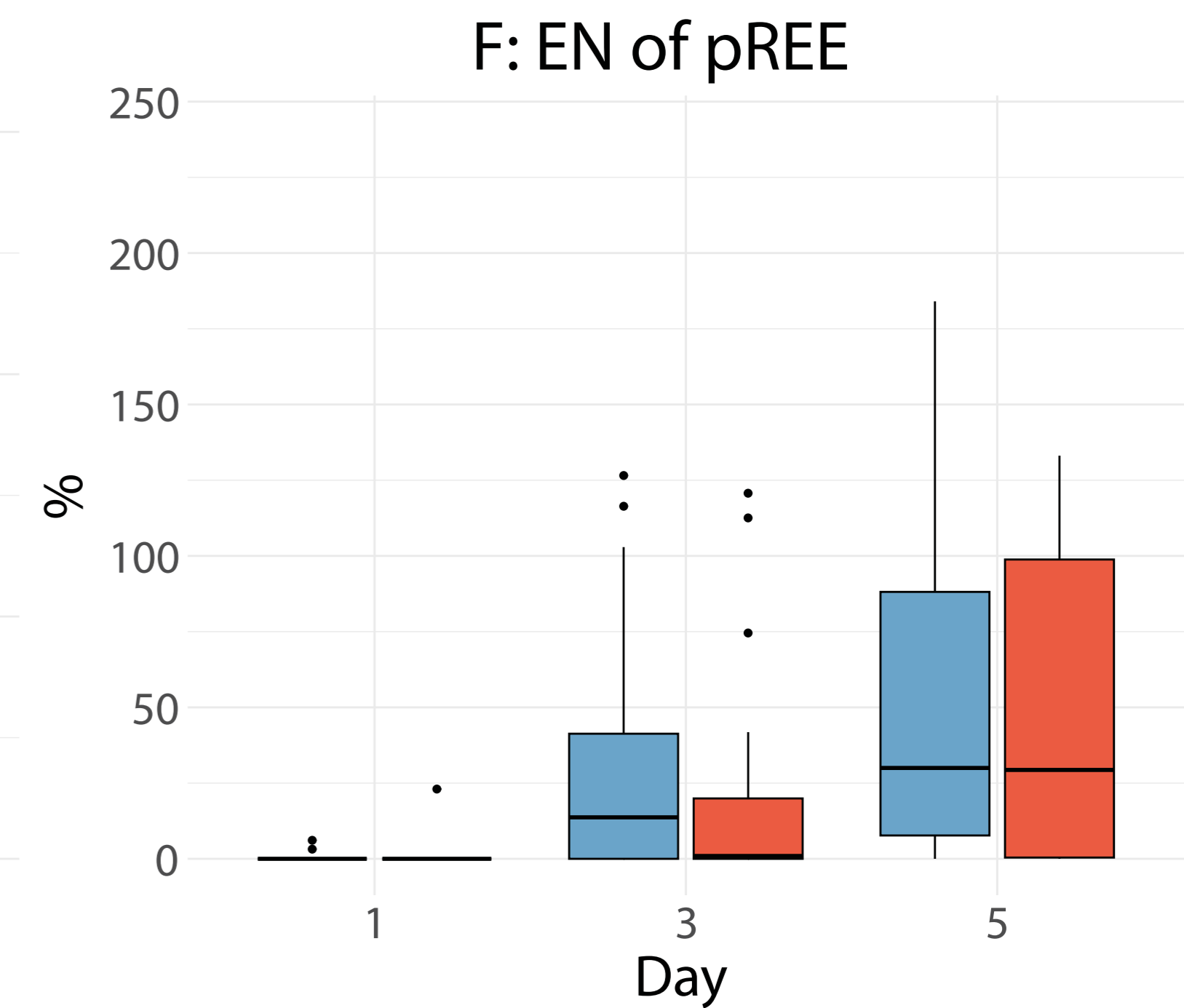

Randomisation    Early-PN    Late-PN

Supplement: Supplementary file 7 [file mpg-77-0811-s007.pdf]
