# Supplementary material for: Gastrointestinal Biomarkers and Their Association with Feeding in the First Five Days of Pediatric Critical Illness
Source: J Pediatr Gastroenterol Nutr. 2023 Sep 20;77(6):811–8. doi: 10.1097/MPG.0000000000003950 (PMC10642702; doi:10.1097/MPG.0000000000003950)
Supplement: Supplementary file 4 [file mpg-77-0811-s004.pdf]

Biomarker	Admission		Day 3		Day 5	
	n	concentration	n	concentration	n	concentration
CCK pg/mL	161	19.1 (14.6; 25.7)	93	28.2 (17.7; 39.4)	66	20.5 (15.0; 25.8)
Leptin µg/L	162	1.5 (0.7; 5.4)	93	2.5 (0.7; 9.6)	64	2.5 (1.3; 7.6)
Glucagon pmol/L	139	0.9 (0.5; 4.5)	79	5.7 (2.3; 12.2)	58	8.9 (3.8; 16.0)
I-FABP2 pg/mL	163	1627 (756; 3491)	94	363 (171; 706)	66	489 (191; 850)
Citrulline µmol/L	97	11.7 (7.7; 17.5)	85	11.8 (8.9; 16.8)	57	12.9 (10.5; 18.5)
% EN of pREE	164	0 (0; 0)	94	14.5 (0.0; 43.8)	66	28.0 (7.6; 94.8)

Table, Supplemental Digital Content 4. The concentrations of the GI biomarkers CCK, leptin, glucagon, I-FABP2, citrulline and the enteral nutrition intake per day for all patients

Concentrations are shown as median (Q1; Q3).

CCK: cholecystokinin, EN: enteral nutrition, I-FABP2: Intestinal fatty-acid binding protein 2, pREE: predicted resting energy expenditure
