# Supplementary material for: Gastrointestinal Biomarkers and Their Association with Feeding in the First Five Days of Pediatric Critical Illness
Source: J Pediatr Gastroenterol Nutr. 2023 Sep 20;77(6):811–8. doi: 10.1097/MPG.0000000000003950 (PMC10642702; doi:10.1097/MPG.0000000000003950)
Supplement: Supplementary file 5 [file mpg-77-0811-s005.pdf]

Biomarker	Day 3			Day 5		
	FIS (n=19)	No FIS (n=75)	P-value	FIS (n=14)	No FIS (n=52)	P-value
**CCK pg/mL**	19.9 (14.0; 28.2)	30.2 (22.2; 40.0)	0.012	23.2 (13.5; 31.2)	20.3 (15.2; 25.6)	0.88
**I-FABP2 pg/mL**	487 (156; 772)	363 (171; 680)	0.95	399 (198; 816)	510 (205; 850)	0.70
**Leptin µg/L**	2.1 (0.7; 2.7)	3.3 (0.7; 10.4)	0.15	2.6 (0.7; 6.9)	2.5 (1.3; 7.8)	0.86
**Glucagon pmol/L**	5.6 (3.3; 8.0)	6.0 (2.2; 15.9)	0.57	3.9 (2.1; 13.7)	9.8 (6.3; 16.1)	0.11
**Citrulline µmol/L**	11.8 (8.2; 18.0)	11.9 (9.0; 16.2)	0.83	17.2 (10.3; 26.2)	12.8 (10.7; 18.3)	0.42
**PYY pg/ml**	9.9 (3.5; 11.2)	8.1 (3.5; 13.6)	0.46	11.5 (7.6; 13.5)	11.9 (7.7; 17.7)	0.42
**% EN of pREE**	12.5 (10.3; 58.0)	16.9 (0; 43.7)	0.31	23.2 (8.7; 27.3)	38.0 (2.6; 101.1)	0.18

Table, Supplemental Digital Content 5. The biomarker concentrations for patients without and with FI symptoms (vomiting/aspiration or large GRV) on admission, day 3, and on day 5, and the statistical differences as tested with the Mann-Whitney U test.

Data are median (Q1; Q3).

CCK: cholecystokinin, EN: enteral nutrition, FIS: feeding intolerance symptoms, I-FABP2: Intestinal fatty-acid binding protein 2, pREE: predicted resting energy expenditure
