# Supplementary material for: Gastrointestinal Biomarkers and Their Association with Feeding in the First Five Days of Pediatric Critical Illness
Source: J Pediatr Gastroenterol Nutr. 2023 Sep 20;77(6):811–8. doi: 10.1097/MPG.0000000000003950 (PMC10642702; doi:10.1097/MPG.0000000000003950)
Supplement: Supplementary file 6 [file mpg-77-0811-s006.pdf]

Characteristic	FIS on day 3 or 5 (n=31)	Never FIS (n=92)
**Randomisation group: Early-PN**	19 (61%)	48 (52%)
**Age in years**	9.3 (7.5; 14.4)	10,6 (4.8; 13,9)
**Sex: Male**	16 (52%)	51 (55%)
**STRONGkids category: high risk**	1 (3%)	9 (10%)
**Diagnostic group**		
**Surgical**		
**Cardiac**	1 (3%)	7 (8%)
**Neuro**	12 (39%)	18 (20%)
**Abdominal**	0 (0%)	5 (5%)
**Other**	8 (26%)	21 (23%)
**Medical**		
**Cardiac**	2 (6%)	6 (7%)
**Neuro**	1 (3%)	9 (10%)
**Respiratory**	3 (10%)	14 (15%)
**Other**	4 (13%)	12 (13%)
**PIM3 score**	-2,6 (-3.1; -1.3)	-2.6 (-3.2; -1.0)

Table, Supplemental Digital Content 6. Baseline characteristics of patients with and without FIS

Data are no. (%) or median (Q1; Q3)

FIS: feeding intolerance symptoms, PEPaNIC: Early versus Late Parenteral Nutrition in the Pediatric Intensive Care Unit, PIM3: Pediatric Index of Mortality, PN: parenteral nutrition, STRONGkids: Screening Tool for Risk on Nutritional Status and Growth score, Q1: first quartile, Q3: third quartile
